# GCAEMDA: Predicting miRNA-disease associations via graph convolutional autoencoder

**DOI:** 10.1371/journal.pcbi.1009655

**Published:** 2021-12-10

**Authors:** Lei Li, Yu-Tian Wang, Cun-Mei Ji, Chun-Hou Zheng, Jian-Cheng Ni, Yan-Sen Su

**Affiliations:** 1 School of Cyber Science and Engineering, Qufu Normal University, Qufu, China; 2 School of Artifial Intelligence, Anhui University, Hefei, China; University of Electronic Science and Technology, CHINA

## Abstract

microRNAs (miRNAs) are small non-coding RNAs related to a number of complicated biological processes. A growing body of studies have suggested that miRNAs are closely associated with many human diseases. It is meaningful to consider disease-related miRNAs as potential biomarkers, which could greatly contribute to understanding the mechanisms of complex diseases and benefit the prevention, detection, diagnosis and treatment of extraordinary diseases. In this study, we presented a novel model named Graph Convolutional Autoencoder for miRNA-Disease Association Prediction (GCAEMDA). In the proposed model, we utilized miRNA-miRNA similarities, disease-disease similarities and verified miRNA-disease associations to construct a heterogeneous network, which is applied to learn the embeddings of miRNAs and diseases. In addition, we separately constructed miRNA-based and disease-based sub-networks. Combining the embeddings of miRNAs and diseases, graph convolutional autoencoder (GCAE) was utilized to calculate association scores of miRNA-disease on two sub-networks, respectively. Furthermore, we obtained final prediction scores between miRNAs and diseases by adopting an average ensemble way to integrate the prediction scores from two types of subnetworks. To indicate the accuracy of GCAEMDA, we applied different cross validation methods to evaluate our model whose performances were better than the state-of-the-art models. Case studies on a common human diseases were also implemented to prove the effectiveness of GCAEMDA. The results demonstrated that GCAEMDA was beneficial to infer potential associations of miRNA-disease.

## Author summary

Numerous studies have demonstrated that miRNAs are closely related to several common human diseases, so observing unverified associations between miRNAs and diseases is conducive to the diagnose and treatment of complex diseases. Considerable models proposed to infer potential miRNA-disease associations have made the prediction more effective and productive. We constructed GCAEMDA model to acquire more accuracy prediction result by integrating graph convolutional network and autoencoder to make prediction based on multi-source miRNA and disease information. The five-fold cross validation and global leave-one-out cross validation were implemented to evaluate the performance of our model. Consequently, GCAEMDA reached AUCs of 0.9415 and 0.9505 respectively that were distinctly higher than AUCs of other comparative models. Furthermore, we carried out case studies on lung neoplasms and breast neoplasms to demonstrate the practical application of the model, 47 and 47 of top-50 candidate miRNAs were confirmed by experimental reports. In summary, GCAEMDA could be considered as an effective and accuracy model to reveal relationship between miRNAs and diseases.

## Introduction

MicroRNAs (miRNAs) belong to one class of significant small endogenous non-coding RNA (~22nt), which inhibit post-transcriptional level of gene expression [1–4]. The first miRNA (lin-4) was observed from the research result of C. elegans larval development timing [5]. The lin-4 functions in a 22 nucleotide regulatory RNA that is different from common protein coding genes [5, 6]. Since the discovery of lin-4, thousands of miRNAs are detected in several living organisms, and then these miRNAs are demonstrated to be associated with many complex biological processes including cell proliferation [7], differentiation [8], development [9], metabolism [10], apoptosis [11] and so on. In addition, miRNAs also are proved to be related to various complex human diseases including heart disease, lung disease, breast disease and so on [12–15]. Hence, the exploration of miRNA-disease association contributes to understanding the molecular mechanisms of different diseases and improving the accuracy of disease diagnosis to a great extent. Considerable traditional experiments have been conducted to infer the relationship between miRNAs and diseases, such as polymerase chain reaction and microarray [16]. But the traditional methods are almost expensive and time-consuming, only little associations of miRNA-disease can be confirmed [17]. Based on the above analyses, it is necessary to propose efficient computational models that can accurately discover interactions of miRNA-disease on a large scale.

According to the theory that functionally similar miRNAs are likely to be allied with phenotypically similar diseases and vice versa [18, 19], numerous computational methods have been proposed to infer possible associations of miRNA-disease. Jiang et al [20] utilized hypergeometric distribution method in the miRNA-disease association prediction model, which combined verified miRNA-disease association data, miRNA similarity matrix and disease similarity matrix to infer unknown interactions between miRNAs and diseases. But the promotion of this model needed to integrate other varieties of biological information, which was laborious to collect and calculate. Wei et al. [21] proposed a computational model to observe the relationship of miRNA-disease by integrating various miRNA similarity information and disease similarity information. After integrating miRNA and disease data sources, they applied kernelized Bayesian matrix factorization to infer unverified miRNA-diseases connections. The model HGIMDA was proposed by Chen et al. [22] that predicted unknown connections by combining similarity networks and the known miRNA-disease association network to construct a heterogeneous graph with all the 3-length paths. However, the shortcoming of HGIMDA was that the best parameters could not be felicitously chosen. Chen et al. [23] applied heterogeneous label propagation algorithm in the HLPMDA model to predict possible relationship of miRNA-disease. They constructed the multi-network of miRNA, long non-coding RNA (lncRNA) and disease to propagate label that could be utilized to infer unknown interaction information of miRNA-disease. Recently, Li et al. [24] predicted miRNA-disease connections by utilizing the modified random forest algorithm that relied on sequence information and symptom information in Seq-SymRF model. Moreover, Euclidean distance-based clustering method was applied to choose reliable negative samples in this model.

Currently, with the amazingly quick development of the intelligence technology, machine learning algorithms are gradually utilized to observe potential relationship between miRNAs and diseases. In order to predict possible miRNAs associated with diseases, Chen et al. [25] utilized within and between score of each miRNA-disease pair in the novel model named WBSMDA. Furthermore, WBSMDA could predict the potential relationship between new diseases and new miRNAs, which had unknown association information. You et al. [26] proposed the prediction model-PBMDA that integrated numerous biological information including known miRNA-disease associations, miRNA functional similarity and Gaussian interaction profile (GIP) kernel similarity, disease semantic similarity and GIP kernel similarity to construct a heterogeneous graph contained three interlinked sub-graphs. Then, depth-first search algorithm was adopted to infer possible miRNA-disease connections. Qu et al. [27] also integrated numerous biological data in MDLPMDA model to predict miRNA-disease associations. They utilized matrix decomposition to process miRNA-disease association matrix for reducing noise data. Then, they applied label propagation method on miRNA similarity network and disease similarity network to obtain different prediction results, respectively. Two different prediction results were combined by an average ensemble way to obtain final target association matrix. Li et al. [28] utilized similarity network fusion method to integrate different kinds of miRNA similarity information and disease similarity information to form final miRNA and disease similarity matrices, respectively. Then, they used inductive matrix completion method on acquired biological data to make an association prediction. Guo et al. [29] utilized multi-layer linear projection method to predict potential miRNA-disease associations. They gradually updated miRNA-disease association matrix by using the top n neighbors of miRNA node and disease node, which contributed to employing the local structure and enhancing data richness. In addition, they utilized updated association matrix, miRNA similarity matrix and disease similarity matrix to construct a heterogeneous matrix, which were utilized by designed multiple computing layers to obtain predicted miRNA-disease association scores.

Meanwhile, there are also a number of graph convolutional network or autoencoder-based methods that are successfully put forward later. For example, the model named HGCNMDA [30] was proposed to infer potential interactions of miRNA-disease. The node2vec method [31] and graph convolutional networks were adopted on Protein-Protein Interactions (PPI) to obtain the cross-features of diseases and miRNAs. HGCNMDA constructed an edge features extraction component based on these cross-features for achieving accuracy predictive performance. The model named NIMCGCN [32] implemented the graph convolutional networks on similarity networks of miRNA and disease for inferring more valuable features. And NIMCGCN applied neural inductive matrix completion to generate predictive miRNA-disease associations. Li et al. [33] proposed the FCGCNMDA model, which applied fully connected homogeneous graph to indicate corresponding correlation coefficient between various miRNA-disease pairs. And then miRNA-disease pairs feature matrix and the fully connected graph were fed into a graph convolutional networks with two-layer for training. Finally, the FCGCNMDA made full use of trained network to infer unknown scores of miRNA-disease pairs. Tang et al [34] developed the MMGCN model to predict potential miRNA-disease associations. The MMGCN severally employed GCN encoder to extract the features of miRNA and disease in different similarity views. Moreover, MMGCN could enhance the acquired latent representations for association prediction by implementing multichannel attention, which adaptively learned the importance of different features. Ji et al. [35] presented the AEMDA model that applied a learning-based way to obtain high-dimensional disease representations and miRNA representations. Then, AEMDA utilized a deep autoencoder that only need positive samples to observe disease-related miRNAs. Li et al. [36] proposed a novel model named GAEMDA that constructed a graph neural networks-based encoder to obtain the miRNA low-dimensional embeddings as well as disease low-dimensional embeddings and achieve the efficacious fusion of heterogeneous information. In addition, the bilinear decoder applied acquired miRNA embeddings and disease embeddings to predict potential associations between miRNAs and diseases. The VAEMDA model [37] utilized integrated miRNA similarity, integrated disease similarity and known miRNA-disease association to construct two spliced matrices, which were used to train the variational autoencoder, respectively. They integrated the prediction scores from different trained variational autoencoder models to infer unverified miRNA-disease associations. Liu et al. [38] developed a computational model called to infer unknown miRNA-disease associations. SMALF first utilized the stacked autoencoder to extract miRNA and disease latent features from the original association matrix of miRNA-disease. Then, SMALF integrated miRNA functional similarity, miRNA latent feature, disease semantic similarity and disease latent feature to form the feature vector of denoting miRNA-disease. Finally, XGBoost was utilized in SMALF to predict potential miRNA-disease associations.

In conclusion, most proposed models first get the feature representations of miRNAs and diseases, and then make prediction with those representations. However, the feature representation can’t completely denote the deep relationships in the network of miRNA-disease, which indicates these models ignore the abundant structural information contained in the network. To overcome the mentioned limitations of existing prediction models, we introduced a novel computational model (GCAEMDA) to infer potential miRNA-disease associations by integrating graph convolutional network and autoencoder. Specifically, the main contributions of our study included following parts:

- The embeddings of miRNAs and diseases were learned by a heterogeneous network, which was constructed by miRNA-miRNA similarity network, disease-disease similarities and known miRNA-disease associations.
- Based on the embeddings of miRNAs and diseases, graph convolution autoencoder was applied to obtain prediction scores of miRNA-disease from miRNA-based and disease-based sub-networks, which were constructed by giving the similarity networks with different threshold values.
- Furthermore, we obtained final prediction scores of miRNA-disease by adopting an average ensemble way to integrate the two prediction scores.

We applied five-fold cross validation (5-CV) and global leave-one-out cross validation (global LOOCV) to evaluate accuracy of the GCAEMDA, which obtained AUCs of 0.9415 and 0.9505, respectively. Case studies on colon neoplasms and prostate neoplasms were also carried out to prove the prediction ability of the model. As a result, most of the predicted miRNAs associated with these diseases were verified by miR2Disease database [39] and dbDEMC v2.0 database [40]. In conclusion, GCAEMDA can effectively predict potential miRNA-disease association.

## Materials

### Human miRNA-disease associations

In this paper, we extracted the verified miRNA-disease association information from HMDD v3.2 database [41] that obtained 12446 experimentally certified associations between 853 miRNAs and 591 diseases after merging duplicates and removing the irregular data. In order to better represent these biological information, we constructed a binary matrix *A*∈*R*^*nm*×*nd*^ to denote known miRNA-disease associations. The *nm* and *nd* were applied to represent the number of miRNAs and number of diseases, respectively. The matrix *A* only included two values that were 1 and 0 indicated verified associations and unverified associations between miRNAs and diseases, respectively.

### miRNA sequence similarity

We obtained corresponding miRNA sequences form miRBase database [42], and the Needleman-Wunsch [43] algorithm was utilized to calculate sequence similarity of miRNAs. For the convenience and efficiency of subsequent calculation, we constructed the matrix *SM*_1_ to store the data. The element *SM*_1_(*m*_*i*_, *m*_*j*_) represents the value of sequence similarity between miRNA *m*_*i*_ and miRNA *m*_*j*_.

### Disease semantic similarity

The MeSH database [44] that contains numerous disease descriptors is obtained by the National Library of Medicine, and disease semantic similarity can be calculated via utilizing the arborescence attribute of disease in MeSH database where every disease node is marked in the directed acyclic graph (DAG) [45]. For a specific disease *D*, we defined *DAG*(*D*) = (*D*,*T*(*D*),*E*(*D*)), where *T*(*D*) denoted the node set that included *D* itself and its ancestor nodes and *E*(*D*) denoted the edge set that included the direct links to connect child nodes with parent nodes directly. We constructed the matrix *SD*_1_ to store the similarity information and element *SD*_1_(*d*_*i*_, *d*_*j*_) represents the value of semantic similarity between disease *d*_*i*_ and disease *d*_*j*_.

### Gaussian interaction profile kernel similarity

On the basis of assumption that the miRNAs with similar functions are likely to be related to diseases with similar phenotypes and vice versa [18, 45], we introduced Gaussian interaction profile kernel similarity to represent miRNA similarity and disease similarity. First, the interaction profile of miRNA *m*_*i*_ was denoted by a binary vector *P*(*m*_*i*_) that represented presence association or absence association between miRNA *m*_*i*_ and each disease in verified miRNA-disease associations, which was also the row *i* of matrix *A*. Furthermore, the GIP kernel similarity of miRNA can be calculated as follows:

$$SM_{2}(m_{i},m_{j})=\exp\left(-\rho_{m}{\left\|P(m_{i})-P(m_{j})\right\|}^{2}\right)$$ (1)

where *SM*_2_(*m*_*i*_, *m*_*j*_) denotes the GIP kernel similarity between miRNA *m*_*i*_ and miRNA *m*_*j*_, and the adjustment parameter *ρ*_*m*_ is defined by the below formula:

$$\rho_{m}=\rho_{m}^{\prime }/(\frac{1}{nm}\sum_{i=1}^{nm}{\left\|P(m_{i})\right\|}^{2})$$ (2)

where $\rho_{m}^{\prime }$ denotes the original bandwidth that is defined as 1 on the basis of the previous study [46].

In the same manner, the GIP kernel similarity *SD*_2_(*d*_*i*_, *d*_*j*_) between disease *d*_*i*_ and disease *d*_*j*_ can be calculated as follows:

$$SD_{2}(d_{i},d_{j})=\exp\left(-\rho_{d}{\left\|P(d_{i})-P(d_{j})\right\|}^{2}\right)$$ (3)

$$\rho_{d}=\rho_{d}^{\prime }/(\frac{1}{nd}\sum_{i=1}^{nd}{\left\|P(d_{i})\right\|}^{2})$$ (4)

### Integrating similarity for miRNA and disease

In this section, the *SM*_1_ and *SM*_2_ are combined to construct the final miRNA similarity matrix *SM* by the below formula.


$$SM(m_{i},m_{j})=\left\{ \begin{array}{l} \frac{{SM}_{1}(m_{i},m_{j})+{SM}_{2}(m_{i},m_{j})}{2}\;if\;{SM}_{1}\left(m_{i},m_{j}\right)\neq 0\\ \;{SM}_{2}(m_{i},m_{j})\;otherwise \end{array}\right.$$ (5)

Similarly, the *SD*_1_ and *SD*_2_ are combined to construct the ultimate disease similarity matrix *SD* by the following formula.


$$SD(d_{i},d_{j})=\left\{ \begin{array}{l} \frac{{SD}_{1}(d_{i},d_{j})+{SD}_{2}(d_{i},d_{j})}{2}\;if\;{SD}_{1}\left(d_{i},d_{j}\right)\neq 0\\ \;{SD}_{2}(d_{i},d_{j})\;otherwise \end{array}\right.$$ (6)

**Fig 1:**
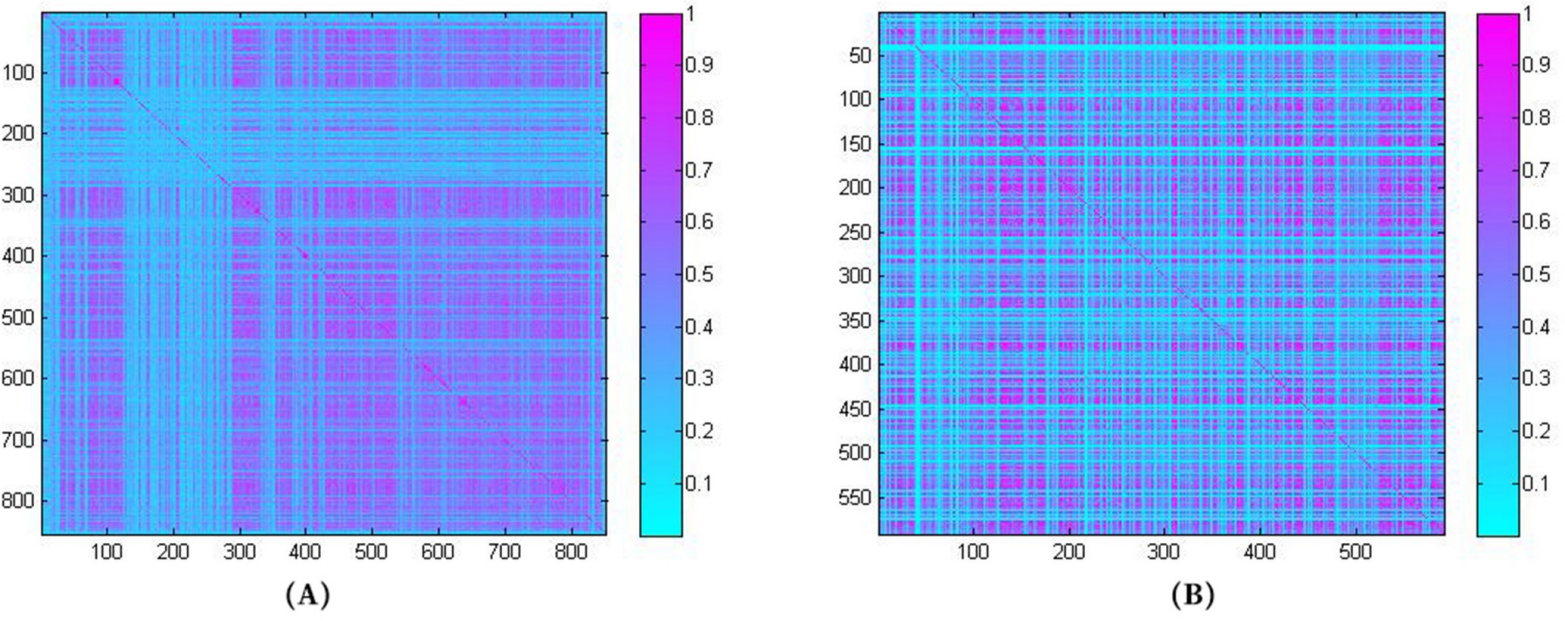
Visualization of (A) miRNA similarity matrix, (B) disease similarity matrix.

In order to clearly show specific information of miRNA similarity matrix and disease similarity matrix, the visualizations of *SM* and *SD* are shown in Fig 1A and 1B.

## Methods

### Heterogeneous network and sub-networks

In order to promote the contribution of similarities in the propagation process of graph convolutional network, we normalized similarity matrices *SM* and *SD* to obtain matrix *M* and matrix *D* by $M={D_{m}}^{-\frac{1}{2}}SM\;{D_{m}}^{-\frac{1}{2}}$ and $D={D_{d}}^{-\frac{1}{2}}{{SD\;D}_{d}}^{-\frac{1}{2}}$, where *D*_*m*_ = *diag*(∑_*j*_*SM*_*ij*_) and *D*_*d*_ = *diag*(∑_*j*_*SD*_*ij*_). Then, we applied the normalized matrices and known human miRNA-disease association matrix *A* to construct a heterogeneous network. The corresponding matrix *X* of heterogeneous network is defined as follow:

$$X=\left[\begin{array}{cc} M & A\\ A^{T} & D \end{array}\right]$$ (7)

where *A*^*T*^ denotes the transpose of adjacent matrix *A*. The matrix *X* can be applied to obtain feature representation of each entity. Specifically, the first *nm* rows of adjacent matrix *X* denote feature vectors of *nm* miRNAs, the feature vector of miRNA *m*_*i*_ can be presented as [*M*_*i*1_, *M*_*i*2_,…,*M*_*inm*_, *A*_*i*1_, *A*_*i*2_,…,*A*_*ind*_]. Similarly, the last *nd* rows of adjacent matrix *X* denote feature vectors of *nd* diseases, the feature vector of disease *d*_*j*_ can be presented as [$A_{j1}^{T},A_{j2}^{T},\ldots ,A_{jnm}^{T},D_{j1},D_{j2},\ldots ,D_{jnd}$].

In addition, we constructed the two sub-networks, which were miRNA-based sub-network and disease-based sub-network, respectively. Specifically, miRNA similarity matrix *SM* and verified miRNA-disease association matrix *A* were utilized to construct miRNA-based sub-network *m*, and we binarized edge-weighted sub-network *m* into unweighted network with the similarity threshold *tm*. As the same way, disease similarity matrix *SD* and verified miRNA-disease association matrix *A* were utilized to construct disease-based sub-network *d*, and similarity threshold *td* was used to binarize edge-weighted sub-network *d* to unweighted network.

### Graph convolutional auto-encoder

**Fig 2:**
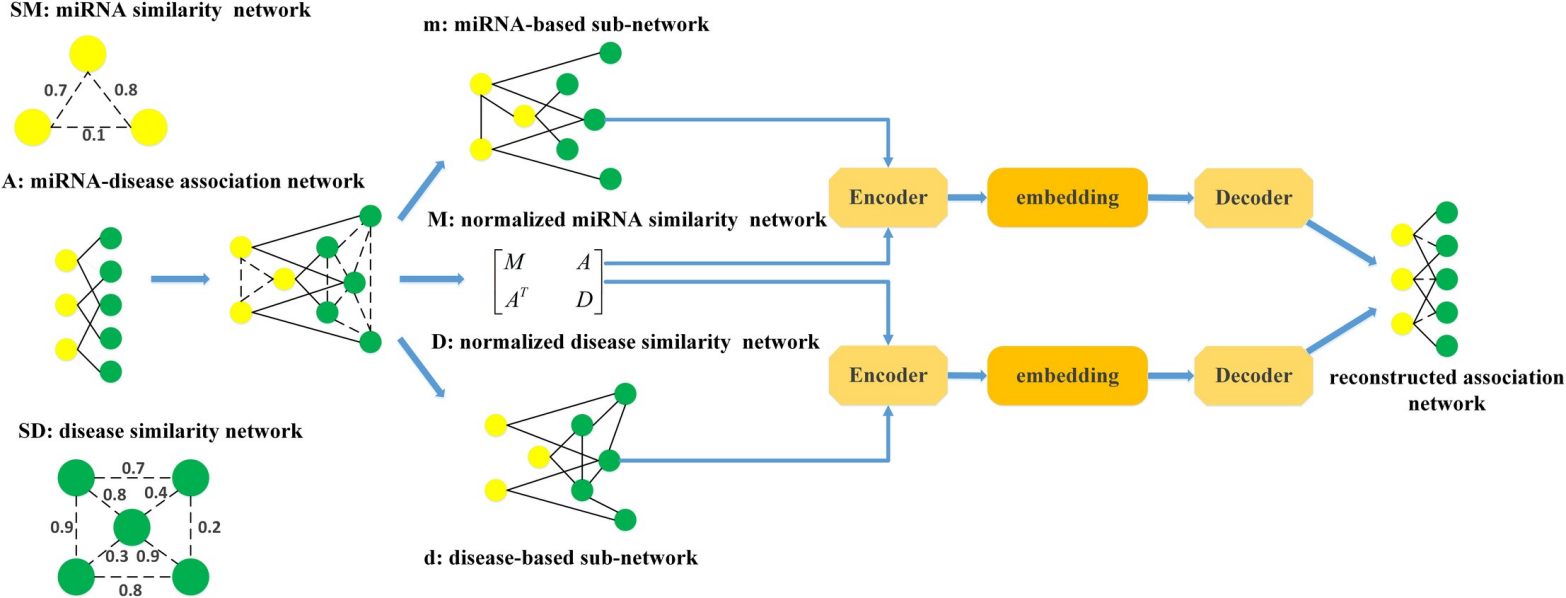
The overview of our proposed GCAEMDA method for predicting potential miRNA-disease association.

The graph convolutional network (GCN) was created by Kipf et al [47], which could effectively learn graph structure information and the representations of node attributes. The graph convolutional auto-encoder (GCAE) can apply GCN to incorporate node features and then utilize latent variables to learn interpretable latent representation for undirected graph from the perspective of data distribution. The GCAE includes graph convolution network encoder and Inner product decoder, which are clearly showed in Fig 2.

GCN encoder contains three layers of graph convolutional networks, which can generate a corresponding lower-dimensional feature matrix. In our paper, we utilized GCN encoder to process miRNA-based sub-network *m* and disease-based sub-network *d*, respectively. The specific process to deal with *m* is shown by following formulas:

$$H^{\left(l+1\right)}=f(H^{\left(l\right)},m)=\sigma (D^{-\frac{1}{2}}\hat{m}D^{-\frac{1}{2}}H^{\left(l\right)}W^{\left(l\right)})$$ (8)

$$D_{ij}=diag(\sum_{j}{\hat{m}}_{ij})$$ (9)

$$\hat{m}=m+I$$ (10)

where *H*^(*l*)^ denotes the embeddings of nodes at the lth layer, *H*^(0)^ is feature matrix *X*, *W*^(*l*)^ denotes a weight matrix which represents a map from high dimensions features to low dimensions features and *σ*(∙) denotes ReLU activation function.

**Fig 3:**
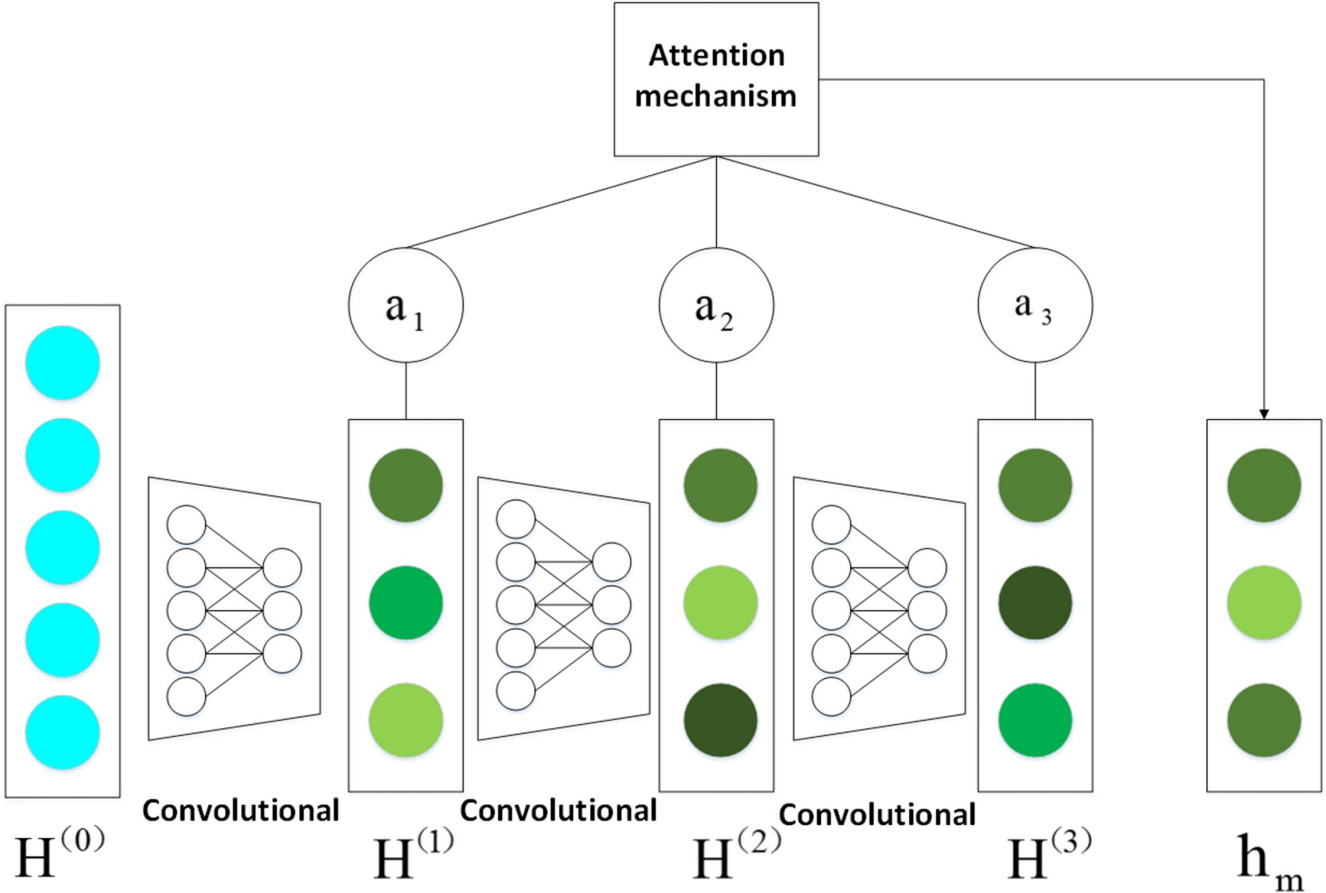
The overview of feature processing in encoder.

Owing to the embeddings of different layers acquire diverse structural information of heterogeneous network, the contributions of these embeddings are inequable. The attention mechanism is introduced to combine embeddings of different layers to obtain final embedding *h*_*m*_ as follows:

$$h_{m}=\sum_{l}a_{l}H^{\left(l\right)}$$ (11)

where *a*_*l*_ represent weight parameters that are utilized to control the contributions of the embeddings at different convolution layers to the final embeddings. In order to better understand above mechanism, the overview of feature processing is clearly shown in Fig 3.

**Fig 4:**
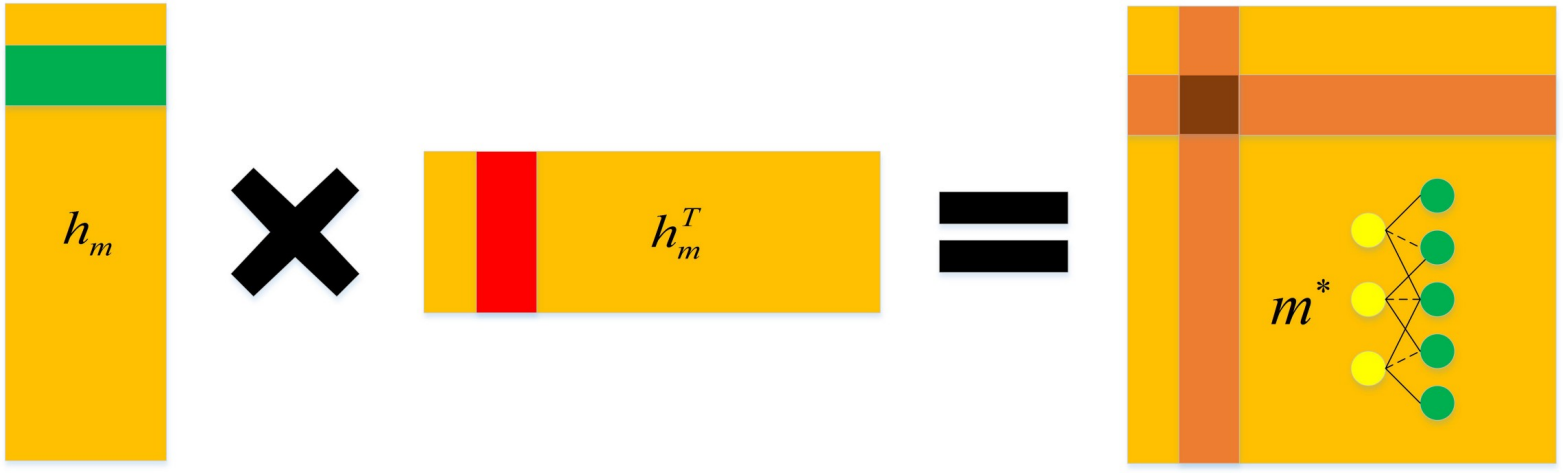
The overview of inner project in decoder.

The decoder in fact is an inner product between latent vector *h*_*m*_, which is briefly showed in Fig 4. According to the principle of matrix factorization (MF), the reconstructed score matrix m* can be calculated by the following formula:

$$m^{*}=sigmoid(h_{m}h_{m}^{T}).$$ (12)

In our paper, we utilized the weighted cross-entropy as loss function, so we should calculate the weighted cross-entropy between the target *m* and the output m*. The loss function is defined as follows:

$$L=-[m*\log\left(sigmoid(m^{*})\right)*\omega +(1-m)*\log\left(1-sigmoid(m^{*})\right)]$$ (13)

where *ω* is weight coefficient, which equals to the radio of negative samples to positive samples.

Similarly, we also used GCN encoder to process disease-based sub-network *d*, and embedding *h*_*d*_ could be obtained, which was final latent vector of disease. In addition, we utilized *h*_*d*_ to obtain reconstructed matrix d*. The weighted cross-entropy between the target *d* and the output d* could be calculated by loss function.

After score matrix m* and score matrix d* were obtained, the ultimate miRNA-disease interaction matrix A* would be get by the average of m* and d*.


$$A^{*}=\frac{m^{*}+d^{*}}{2}$$ (14)

## Results

### Experiment setting

GCAEMDA is conducted on Python based on PyTorch that is considered as an open source machine learning framework, and version 1.1 is implemented in our experimental environment. In addition, the Ubuntu 16.04 platform with 2 Tesla P100 GPUs is used to run our all experiments.

In this study, several hyperparameters more or less affect the performance of the GCAEMDA. The model includes miRNA-based sub-network and disease-based sub-network, which are kept the same structure. Adam optimizer is chosen as the optimizer during the training, and the Relu function [47] is chosen as active function in all hidden layers. The learning rate, number of layers and dimensionality of embeddings ultimately are set to 1*e*−4, 3 and 64 after many adjustments, respectively. We considered different combinations of the total training epochs of GCAEMDA *β* and the dropout rate *γ* from the ranges *β*∈{100, 200, 300, 400} and *γ*∈{0.1, 0.2, 0.3, 0.4, 0.5, 0.6}. As a result, we set *β* = 400 and *γ* = 0.3 for reaching the best experimental results.

### Performance comparison

In this section, we compared GCAEMDA with other excellent computational methods that included BNPNDA [48], MSCHLMDA [49], NIMCGCN [32] and HFHLMDA [50] by implementing 5-CV and global LOOCV. In order to make comparative results persuasive, we used biological information consistent with GCAEMDA in compared methods.

**Fig 5:**
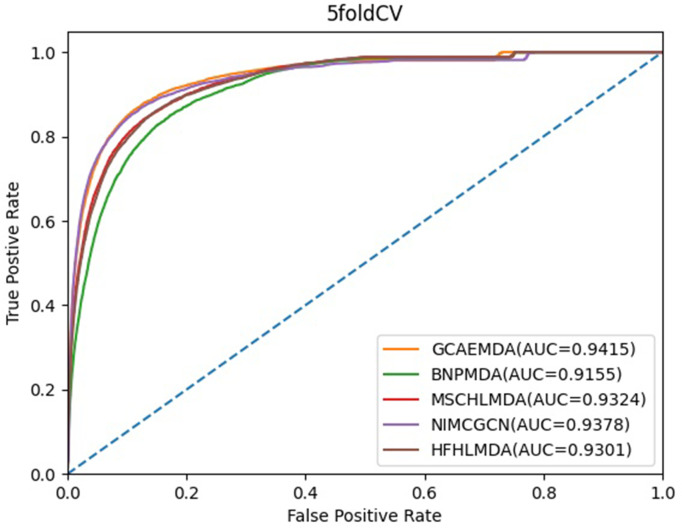
AUC of 5-CV compared with those of BNPMDA, MSCHLMDA, NIMCGCN and HFHLMDA.

In the framework of 5-CV, we divided verified associations into five folds in a random way, and test set was held by each fold in turn, training set included rest parts. And many times repeated segmentations on verified positive samples were applied to reduce potential deviations. In order to effectively evaluate the performance of these models, we calculated the areas under the Receiver operating characteristics (ROC) curves (AUCs) of these methods whose values were between 0 and 1. In the ROC curve, the false positive rate (FPR) and true positive rate (TPR) are served as horizontal axis and vertical axis, respectively. As shown in Fig 5, we could see that GCAEMDA, BNPMDA, MSCHLMDA, NIMCGCN and HFHLMDA acquired AUC values of 0.9415, 0.9155, 0.9314, 0.9378 and 0.9301 in 5-CV.

**Fig 6:**
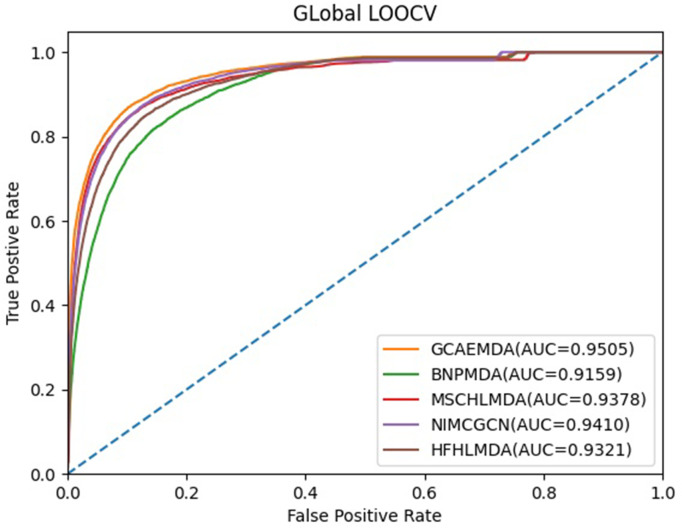
AUC of global LOOCV compared with those of BNPMDA, MSCHLMDA, NIMCGCN and HFHLMDA.

In the framework of global LOOCV, the test set was orderly hold by each verified association, the training set consisted of other verified associations. Once again the AUCs of these models were applied to reflect their performance. As shown in Fig 6, we could see that GCAEMDA, BNPMDA, MSCHLMDA, NIMCGCN and HFHLMDA acquired AUC values of 0.9505, 0.9159, 0.9378, 0.9410, and 0.9321, respectively, which were significantly demonstrated the performance of GCAEMDA was better than other comparative models.

## Performance analysis

Because similarity thresholds *tm* and *td*, which are used to convert edge-weighted similarity network into unweighted network for generating sub-networks, determine the number of miRNA similarities and number of disease similarities, they can affect the prediction performance of model. In this section, we specifically analyze the values of *tm* and *td*.

**Fig 7:**
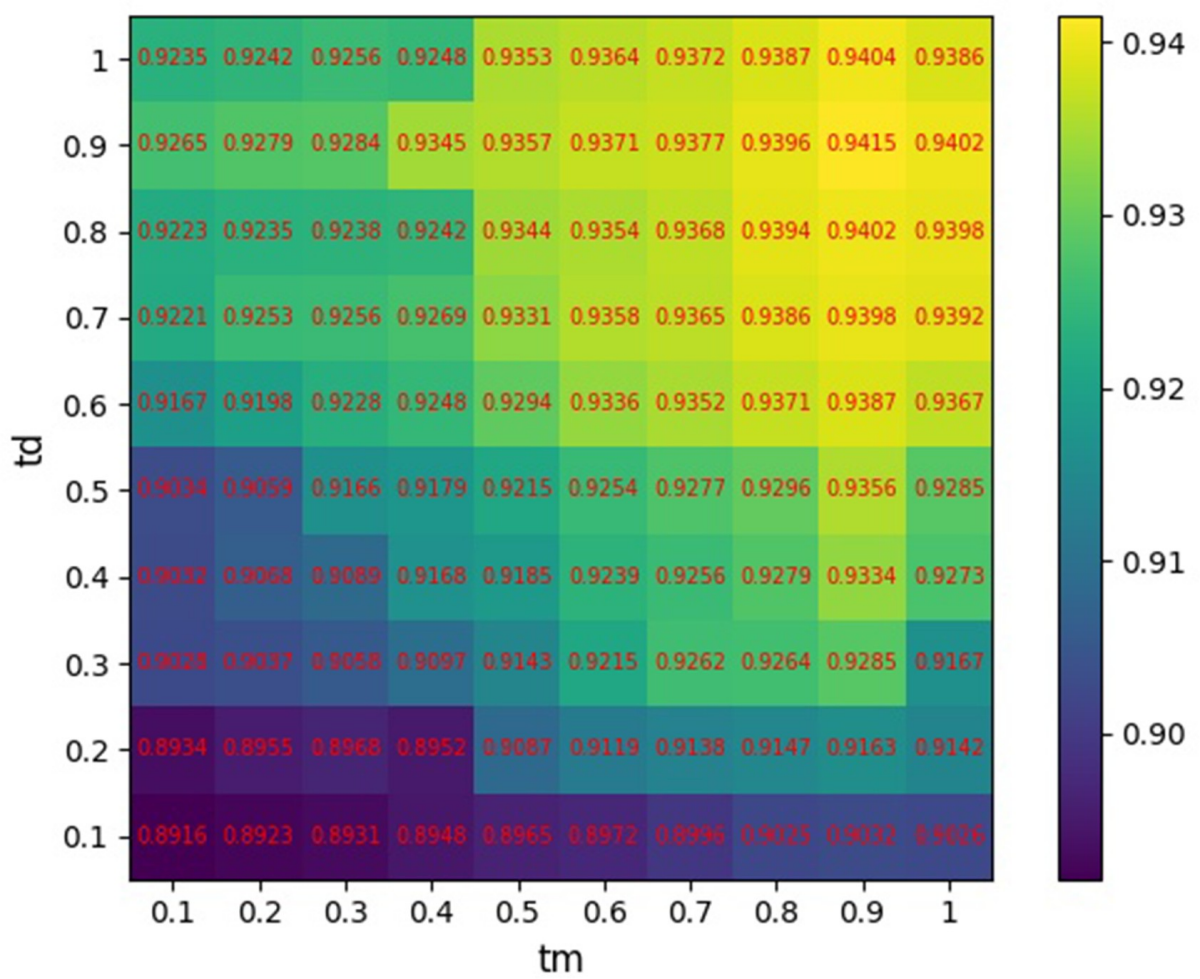
AUCs of 5-CV at different values of *tm* and *td*.

The *tm* and *td* are set to different values as {0.1, 0.2,…,0.9, 1}. The AUC of 5-CV is applied to evaluate the prediction performance, and the influence of different combinations of *tm* and *td* on the prediction results of GCAEMDA under 5-CV is shown in Fig 7. It is obvious that when the values of *tm* and *td* are chose as 0.9 and 0.9, respectively, the model obtains the best prediction performance. Furthermore, we could draw a conclusion that the influence of parameter *tm* on the prediction performance of model is greater than the influence of parameter *td* by analyzing experiment results, which may be attribute to the number of miRNAs nodes is more than the number of diseases nodes.

## Layer attention mechanism analysis

Layer attention is regarded as one important component of the network architecture in encoder and plays an important role in controlling and quantifying the inter-dependence of distinct convolution layers. In this section, we concluded the influence of the layer attention mechanism.

**Fig 8:**
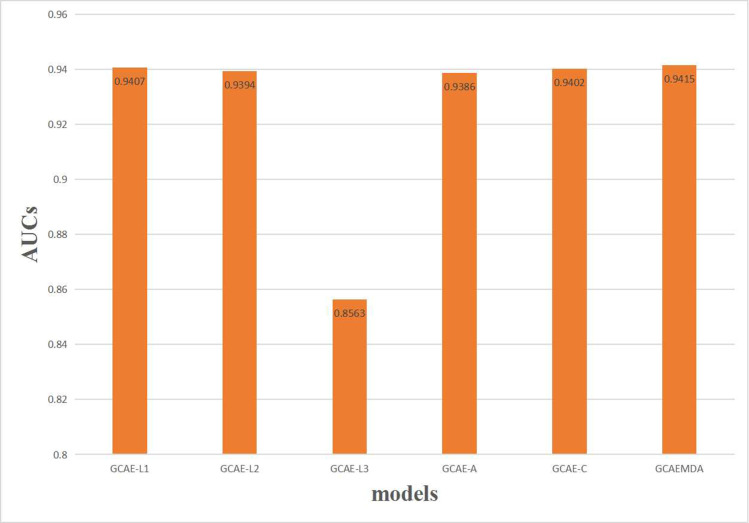
Performance of GCAEMDA based on different embeddings.

We utilized the embeddings at the *l*-th layer of encoder with *l* = 1, 2, 3 to build different models, which are abbreviated as GCAE-L1, GCAE-L2 and GCAE-L3, respectively. The AUCs of these models obtained by the 5-CV are shown in Fig 8, which clearly indicates the prediction performances of GCAE-L1 and GCAE-L2 are better than prediction performance of GCAE-L3. The results denote that the node information contained by first-layer embeddings and second-layer embeddings is more than the third-layer embeddings, which may be attributed to the over-smoothing of GCN [48]. GCAEMDA integrates embeddings of all layers to obtain the prediction result that is better than the prediction results of GCAE-L1, GCAE-L2 and GCAE-L3.

**Fig 9:**
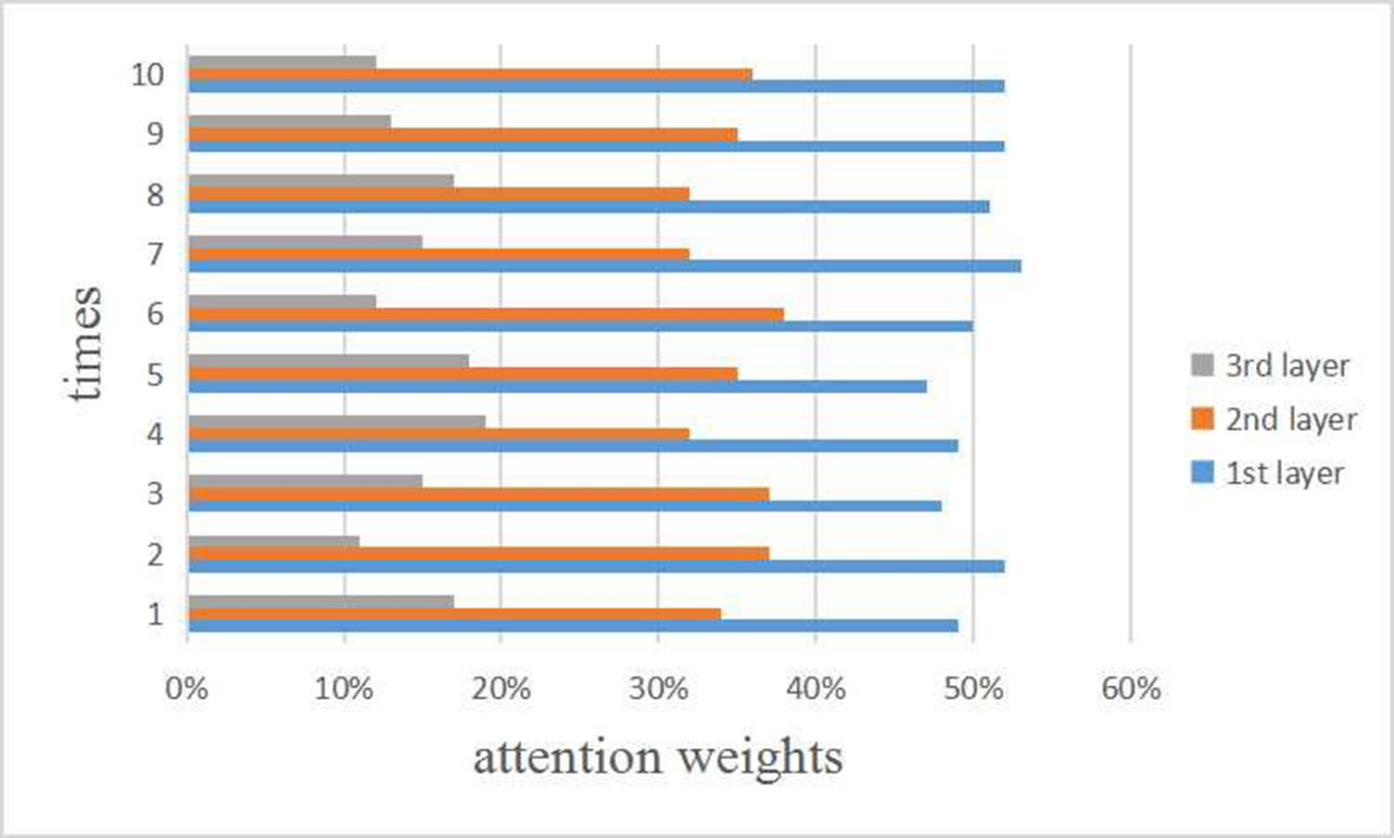
Attention weights for three convolution layers in GCAEMDA.

On the basis of previous study [51], the *l*-th layer of GCN can capture the *l*-th order proximity, so the attention weight indicates the corresponding contribution of the embeddings at every convolution layer to the ultimate embeddings. The 5-CV was implemented in 10 times for GCAEMDA, and the attention weights for three layers based on the computational results were visualized in Fig 9. Three layers possess different attention weights and gradually decreases, which meets the condition in GCN that the higher order, the lower contribution [51]. The results also contribute to state the performances of GCAE-L1, GCAE-L2 and GCAE-L3 in Fig 8. Hence, paying different attention weights to three convolution layers is conducive to improve prediction performance. According to experiment results, we paid 50%, 35% and 15% attention weights to *l*-th layer with *l* = 1, 2, 3, respectively.

There are also other ways to combining embeddings at different layers, so we use GCAE-A and GCAE-C to compare with our model for demonstrating the effectiveness of attention mechanism in GCAEMDA. GCAE-A assigns same weights to different embeddings; GCAE-C directly concatenates different embeddings. The comparison results are also shown in Fig 8, which indicates GCAEMDA produces better result than GCAE-A and GCAE-C.

### Case studies

For the purpose of demonstrating the effectiveness and accuracy of GCAEMDA, we applied the case studies on lung neoplasms and breast neoplasms to validate the performance of GCAEMDA for new miRNA-disease association prediction. Lung neoplasms have long been considered as the leading cause of death worldwide [52]. And breast neoplasms are considered as the common female cancer whose molecular mechanisms should be thoroughly exploited for better treatment [53]. Hence, there is an increasing requirement for seeking biomarkers that can strengthen biological mechanisms understanding of lung neoplasms and breast neoplasms. In this section, all verified associations in the HMDD v3.2 database were put into the training set of GCAEMDA. Then, according to their prediction scores, the top 50 investigated disease-related miRNAs could be selected. Furthermore, we would utilize miR2Disease and dbDEMC v2.0 databases to verify these findings. The miR2Disease database was confirmed manually after the automatic extraction. The dbDEMC database collected differentially expressed miRNAs in various human diseases, and dbDEMC v2.0 added more disease-related miRNAs obtained from expression data. The results demonstrated that a part of verified miRNA-disease associations in HMDD v3.2 database also existed in miR2Disease and dbDEMC v2.0 databases after comparing the HMDD v3.2 with miR2Disease/ dbDEMC v2.0. As a result, 47 out of the top 50 miRNAs were verified to be associated with lung neoplasms (Table 1), and 47 out of the top 50 miRNAs were verified to be associated with breast neoplasms (Table 2). The results demonstrated that our model could effectively predict unknown miRNA-disease associations.

**Table 1:** The top 50 potential miRNAs associated with lung neoplasms.

miRNA	evidence	miRNA	evidence
hsa-mir-93	m; d	hsa-mir-660	d
hsa-mir-96	d	hsa-mir-610	d
hsa-mir-99a	m; d	hsa-mir-545	d
hsa-mir-98	m; d	hsa-mir-616	d
hsa-mir-7	m; d	hsa-mir-671	d
hsa-mir-31	m; d	hsa-mir-605	d
hsa-mir-9	m; d	hsa-mir-520h	d
hsa-mir-574	d	hsa-mir-608	d
hsa-mir-630	d	has-mir-205	m; d
hsa-mir-570	d	hsa-mir-557	d
hsa-mir-95	m; d	hsa-mir-526b	d
hsa-mir-592	d	hsa-mir-522	d
hsa-mir-92a	d	hsa-mir-564	d
hsa-mir-629	d	hsa-mir-1298	d
hsa-mir-939	d	hsa-mir-1973	d
hsa-mir-638	d	hsa-mir-3662	d
hsa-mir-944	d	hsa-mir-412	d
hsa-mir-652	d	hsa-mir-4302	d
hsa-mir-769	unconfirmed	hsa-mir-4326	d
hsa-mir-558	d	hsa-mir-4423	unconfirmed
hsa-mir-548j	unconfirmed	hsa-mir-4500	d
has-mir-614	d	has-mir-1323	d
hsa-mir-641	d	hsa-mir-143	m; d
hsa-mir-937	d	hsa-mir-145	m; d
hsa-mir-873	d	has-mir-21	m; d

m: miR2Disease database; d: dbDEMC v2.0 database

**Table 2:** The top 50 potential miRNAs associated with breast neoplasms.

miRNA	evidence	miRNA	evidence
hsa-mir-96	m; d	hsa-mir-519d	d
hsa-mir-93	d	hsa-mir-718	d
hsa-mir-99a	d	hsa-mir-663a	d
hsa-mir-542	d	hsa-mir-760	d
hsa-mir-92b	d	hsa-mir-561	d
hsa-mir-98	m; d	hsa-mir-608	d
hsa-mir-630	d	hsa-mir-627	d
hsa-mir-708	d	hsa-mir-98	m
hsa-mir-625	d	has-mir-520h	d
hsa-mir-574	unconfirmed	hsa-mir-873	d
hsa-mir-629	d	hsa-mir-570	d
hsa-mir-590	unconfirmed	hsa-mir-603	d
hsa-mir-663	m	hsa-mir-584	d
hsa-mir-9	m; d	hsa-mir-888	d
hsa-mir-874	d	hsa-mir-576	unconfirmed
hsa-mir-520c	m	hsa-mir-661	m; d
hsa-mir-613	d	hsa-mir-605	d
hsa-mir-675	d	hsa-mir-573	d
hsa-mir-638	d	hsa-mir-526b	d
hsa-mir-940	d	hsa-mir-519e	d
hsa-mir-592	d	hsa-mir-765	d
has-mir-650	d	has-mir-575	d
hsa-mir-744	d	hsa-mir-942	d
hsa-mir-939	d	hsa-mir-618	d
hsa-mir-671	d	has-mir-548c	d

m: miR2Disease database; d: dbDEMC v2.0 database

## Discussion and conclusion

In this paper, we introduced a novel method of GCAEMDA in which we applied graph convolutional autoencoder to predict possible associations between miRNAs and diseases. In this model, we applied miRNA sequence similarity and GIP kernel similarity to integrate miRNA similarity network as well as applied disease semantic similarity and GIP kernel similarity to integrate disease similarity network. Then, we utilized miRNA similarity network, disease similarity network and verified miRNA-disease interaction network to construct the heterogeneous network that was applied to obtain the embeddings of miRNAs and diseases. In addition, graph convolution auto-encoder was applied to predict unknown miRNA-disease associations by utilizing the embeddings of miRNAs and diseases as well as miRNA-based and disease-based sub-networks, respectively. Furthermore, we obtained final prediction scores of miRNA-disease by integrating the two prediction score matrix. In the frameworks of 5-CV and global LOOCV, the AUCs of GCAEMDA achieved 0.9415 and 0.9505 that indicated the performance of GCAEMDA had a significant improvement relative to previous methods. And the case studies implemented on lung neoplasms and breast neoplasms also confirmed the prediction ability of GCAEMDA. In conclusion, all results demonstrated that GCAEMDA could effectively observe disease-associated miRNAs.

What should be denoted is that the following factors may contribute to the reliable performance of GCAEMDA. First of all, we integrated plentiful biological data to construct the heterogeneous network that ensured the information richness of embeddings of miRNAs and diseases. In addition, we adaptively combined embeddings at different convolution layers with an attention mechanism, which leads to a more information representation of miRNAs and diseases. Moreover, we constructed miRNA-based and disease-based sub-networks that guaranteed the full completion of missing data.

However, there are some limitations that may influence the performance of GCAEMDA. The data we utilized included verified interactions of miRNA-disease, miRNA similarity data as well as disease similarity data, which may obtain noise and outliers. In addition, GCAEMDA is possible to cause several bias to diseases that are related to plenty of miRNAs and vice versa. Therefore, we should continuously optimize our model to improve its performance in the later days.

## Supporting information

S1 Table Known human miRNA-disease associations obtained from HMDD v3.2 database. (XLSX)

S2 Table Names of 853 miRNAs involved in known human miRNA-disease associations obtained from HMDD v3.2 database. (XLSX)

S3 Table Names of 591 diseases involved in known human miRNA-disease associations obtained from HMDD v3.2 database. (XLSX)

S4 Table The constructed miRNA sequence similarity score matrix. (XLSX)

S5 Table The constructed disease semantic similarity score matrix. (XLSX)
